# Development and validation of a species-specific environmental DNA (eDNA) primer for endangered Asian arowana, *Scleropages formosus* (Teleostei: Osteoglossidae) for law enforcement and wild population monitoring

**DOI:** 10.1016/j.mex.2024.103133

**Published:** 2024-12-31

**Authors:** Nurul Affiqah Edora Mohd-Azhar, Haslawati Baharuddin, Mohamad-Sufiyan Salmi, Noor Faizah Ismail, Amatul-Samahah Md. Ali, Syazwan Saidin, Adibah Abu-Bakar

**Affiliations:** a Department of Biology, Faculty of Science and Mathematics, Universiti Pendidikan Sultan Idris (UPSI), Tanjong Malim, Perak 35900, Malaysia; b Fisheries Research Institute (FRI) Glami Lemi, Jelebu, Negeri Sembilan 71650, Malaysia

**Keywords:** Species-specific primer, Cytochrome oxidase I, Conservation, Aquarium trade, Wildlife monitoring, Development of species-specific eDNA primer for the detection of the Asian arowana (*Scleropages formosus*)

## Abstract

The Asian Arowana, *Scleropages formosus* (Müller and Schlegel, 1844) is a large majestic freshwater teleost, crowned as the king of aquariums with its bright charismatic appearance and magnificent swimming performance. The most expensive Asian arowana is the Golden Blue-based Malayan Arowana which is endemic to the Bukit Merah Lake and Kerian River Basin, Perak, Malaysia. *S. formosus* has been listed as endangered by the IUCN (International Union for Conservation of Nature), regulated under Appendix 1 of the Convention of International Trade on Endangered Species (CITES) for commercial trade. Environmental DNA (eDNA) analysis has become widely accepted in biodiversity monitoring for the detection of rare and endangered species without harming any ecosystem or threatened species. Hence, the application of eDNA as wild population monitoring of *S. formosus* is possible for conservation and CITES enforcement program.

• The species-specific primer of *S. formosus* was designed based on selected sequences obtained from GenBank
• This report presents the potential application of eDNA in the management of the Malaysian 686 CITES Act for conservation monitoring of the Asian Arowana
• The detection and wild population monitoring is possible through the eDNA method as complementary tools

## Graphical abstract

Workflow of development of eDNA application for detection and monitoring of the endangered wild Asian arowana, *Scleropages formosus*

**Figure:**
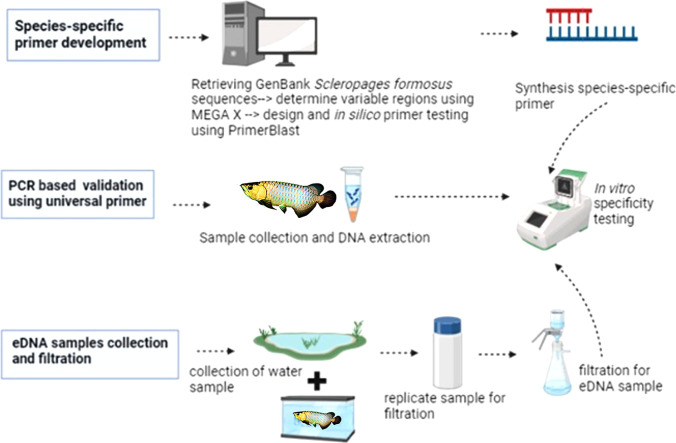

Specifications table

**Table:** 

Subject area:	Agricultural and Biological Sciences
More specific subject area:	Fish biodiversity research and monitoring using eDNA barcoding
Name of your method:	Development of species-specific eDNA primer for the detection of the Asian arowana (*Scleropages formosus*)
Name and reference of original method:	• S.M. Baillie, C. McGowan, S. May-McNally, B.J.G. Sutherland, S. Robinson, K. Street. (2019). Environmental DNA and its applications to Fisheries and Oceans Canada: national needs and priorities, Can. J. Fish. Aquat. Sci., 3329 (2019) • T. Minamoto, M. Miya, T. Sado, S. Seino, H. Dol, M Kondoh, et al., An illustrated manual for environmental DNA research: Water sampling guidelines and experimental protocols, Environ. DNA. 121 (2021) 1-13. • NF Nabilah, AB Adibah, AR Ramizah, S Syazwan, AG Intan-Faraha, A Amirrudin, MN Siti Azizah, Development of species-specific Cichla species eDNA primers for rapid alien invasive species (AIS) monitoring, Tropical Gene., 2(1) (2022) 12-16. • AJ Sepulveda, NM Nelson, CL Jerde, G Luikart, Are environmental DNA methods ready for aquatic invasive species management? Trends Ecol Evol. 35 (2020) 668–678.
Resource availability:	Information on all resources needed to reproduce this procedure is included in the present article.

## Method details

### Species -specific primer development and in silico test

Reference sequences that target the mitochondrial cytochrome c oxidase I (COI) gene were retrieved from GenBank (www.ncbi.nlm.nih.gov) to develop a species-specific primer for *S. formosus*. The acquired sequences were aligned using MEGA X [1] and the results were analysed using BLAST [2] to determine the degree of similarity and difference between them. The found variable areas served as possible sites to develop species-specific primers using the NCBI GenBank's Primer-BLAST program (www.ncbi.nlm.gov/tools/primer-blast) [3].

To achieve species specificity, we looked for primer sequence targets that differed by no more than two base pairs (bp) from non-target species primer sequences, using the criteria from [4]. Each hypothetical primer was submitted to an *in silico* test using the Primer-BLAST tool's default parameters [3]. The process was created as stringent as feasible to avoid the unintended target. A primer pair is termed specific if no amplicons are detected on any targets other than the *S. formosus* species sequence template. Otherwise, it is viewed as nonspecific.

Five species-specific primer pairs, designated as S1, S2, S3, S4, and S5, were developed. All specified primer pairs are 18 to 23 bp long, with an annealing temperature of 50°C and a predicted amplified product of 100 to 150 bp. Details of these primers are in Table 1.

**Table 1:** Information of developed species-specific primers for *S. formosus* for this study.

Name of Primer pair	Primer sequence (5’-3’)	Min GC(%)	Max length (base)	Amplicon size (bp)
S1	F:GACTGCCCTCAGCCTCCTAAA R:TAGCCAAAGCGGCCAATTAAA	55.48 56.22	21 21	100
S2	F:TACTGCGCTCAGCCTCCTA R:GAGCCAAAGCCCCCAATTA	55.23 56.41	19 19	125
S3	F:TACTGCCCTCAGCCTCCTA R:TTGCCAAAGCCCCCAATTA	57.89 47.37	19 19	115
S4	F:TACTGCAATCAGCCTCCTAA R:TTGCCTTCGCCCCCAATTAA	54.31 47.33	20 20	133
S5	F:ATCTGCCCTCAGCCTCCTA R:TTGCCATTGCCGCCAATTA	55.21 56.46	19 19	150

Denotation F: forward primer, R: reverse primer

### Sample collection and DNA extraction

Asian arowana scale samples were carefully taken from the live specimens kept in the cages of the proposed Arowana Sanctuary of Bukit Merah Lake, under monitoring by the Department of Fisheries, Malaysia. The samples were photographed, and assigned with a lab inventory number. The samples were photographed, and a lab inventory number was assigned. Genomic DNA was extracted using the DNEasy Blood and Tissue Kit (Qiagen, USA) according to the manufacturer's instructions, with some modifications. Eluted DNA was measured using the UV spectrophotometer Q3000 (Quawell, USA).

### PCR-based *in vitro* specificity testing

**Fig. 1:**
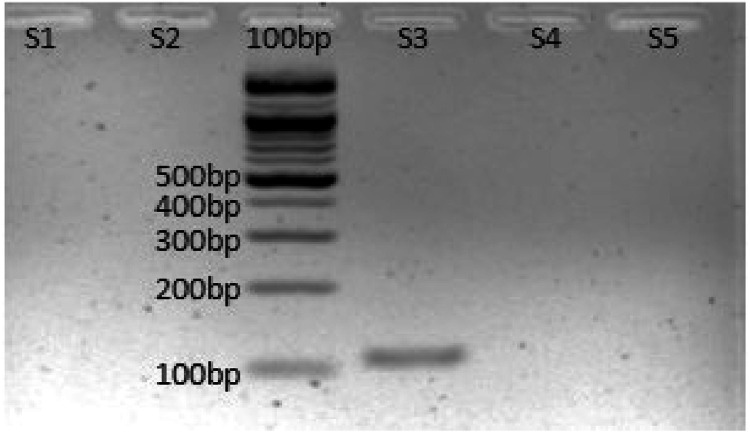
*In vitro* testing for the five developed primers against extracted genomic DNA of *S. formosus*.

Genomic DNA extracted from scale samples from the section above was used to validate the developed primers of *S. formosus*. Among the five primers developed, only one primer, S3 has successfully produced an intense single band for *S. formosus* (Fig. 1) after three replicates of *in vitro* specificity testing.

Negative control samples from four Malaysian native fishes, *Puntioplites bulu* (N1), *Channa micropeltes* (N2), *Thynnichthys thynnoides* (N3), and *Osteochilus hasseltii* (N4), were used to test and verify the primer specificity. The fish genomic DNA samples were amplified using the universal DNA barcoding COI gene primer F1 [FishF1-5′-TCAACCAACCACAAAGACATTGGCAC-3′ and R2 [FishR2-5′-ACTTCAGGGTGACCGAAGAATCAGAA-3′] by [5]. Amplification was done in a 25 µL PCR reaction mixture containing 2X EconoTaq PLUS PCR Master Mix solution (Sigma-Aldrich, Germany), 0.4 µM for both forward and reverse primer, using DNA template (120-500 ng) with molecular-grade water as a negative control. Following that, the N1, N2, N3, and N4 samples, together with *S. formosus* (SF) genomic DNA, were amplified with the S3 primer to confirm its species-specificity.

PCR reactions were carried out in a thermal cycler (T100 Bio-Rad, USA) with an initial denaturation step at 95°C for 2 minutes, 35 cycles at 94°C for 30 seconds, an annealing step at the temperature settings 50°C for 45 seconds, an elongation step at 72°C for 90 seconds and a final extension step at 72°C for 5 minutes. The PCR product (5 µL) was examined using 2% agarose gel electrophoresis with 1 uL GelRed dye (Sigma-Aldrich, Germany). The gel image was captured using GeneSnap software (Syngene, UK).

**Fig. 2:**
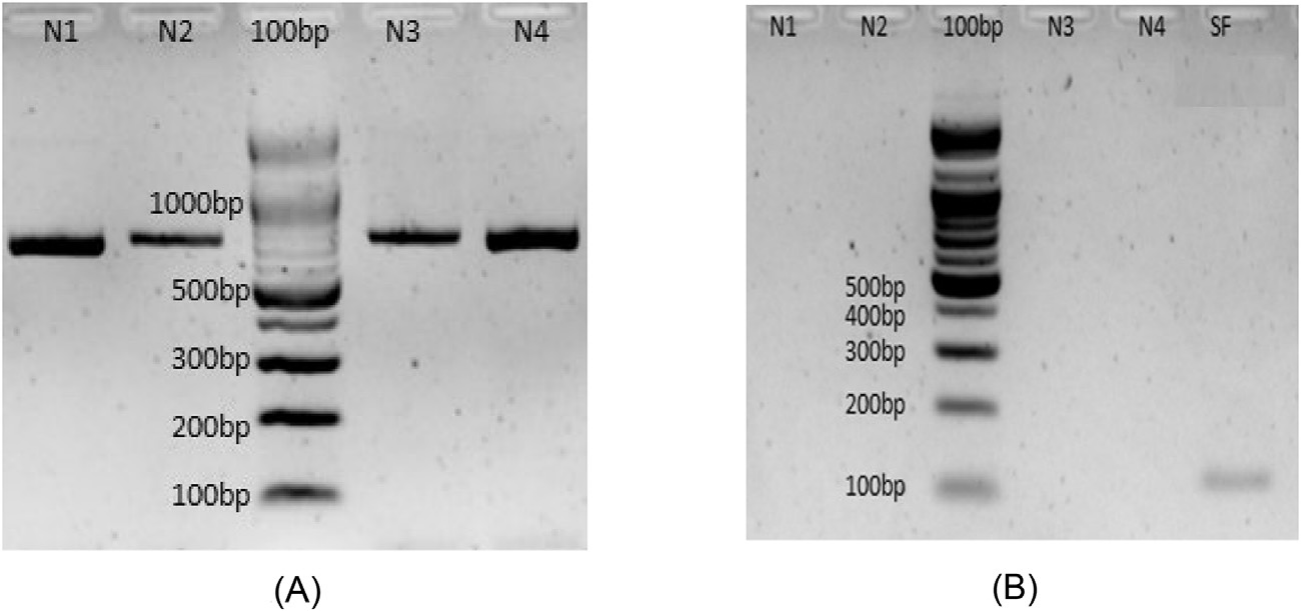
(A): Amplification of N1-N4 local fish genomic DNA using universal COI gene; 2(B): Primer test PCR showing the positive amplification of *S. formosus* (SF) against comparative samples (N1-N4) suggesting the specificity of the designed primer.

In Fig. 2(A), all native samples (N1-N4) showed clear bright bands of high-quality amplification, of the extracted DNA. Following that, the N1-N4 samples together with *S. formosus* (SF) genomic DNA were amplified using S3 primer to verify the primer species-specificity. As shown in Fig. 2(B), a distinct band of around 123 bp in size was detected for the SF sample. There was no amplification for the other four samples, demonstrating the S3 primer specificity for *S. formosus*.

### eDNA samples collection from lake water (field samples) and positive controls

**Fig. 3:**
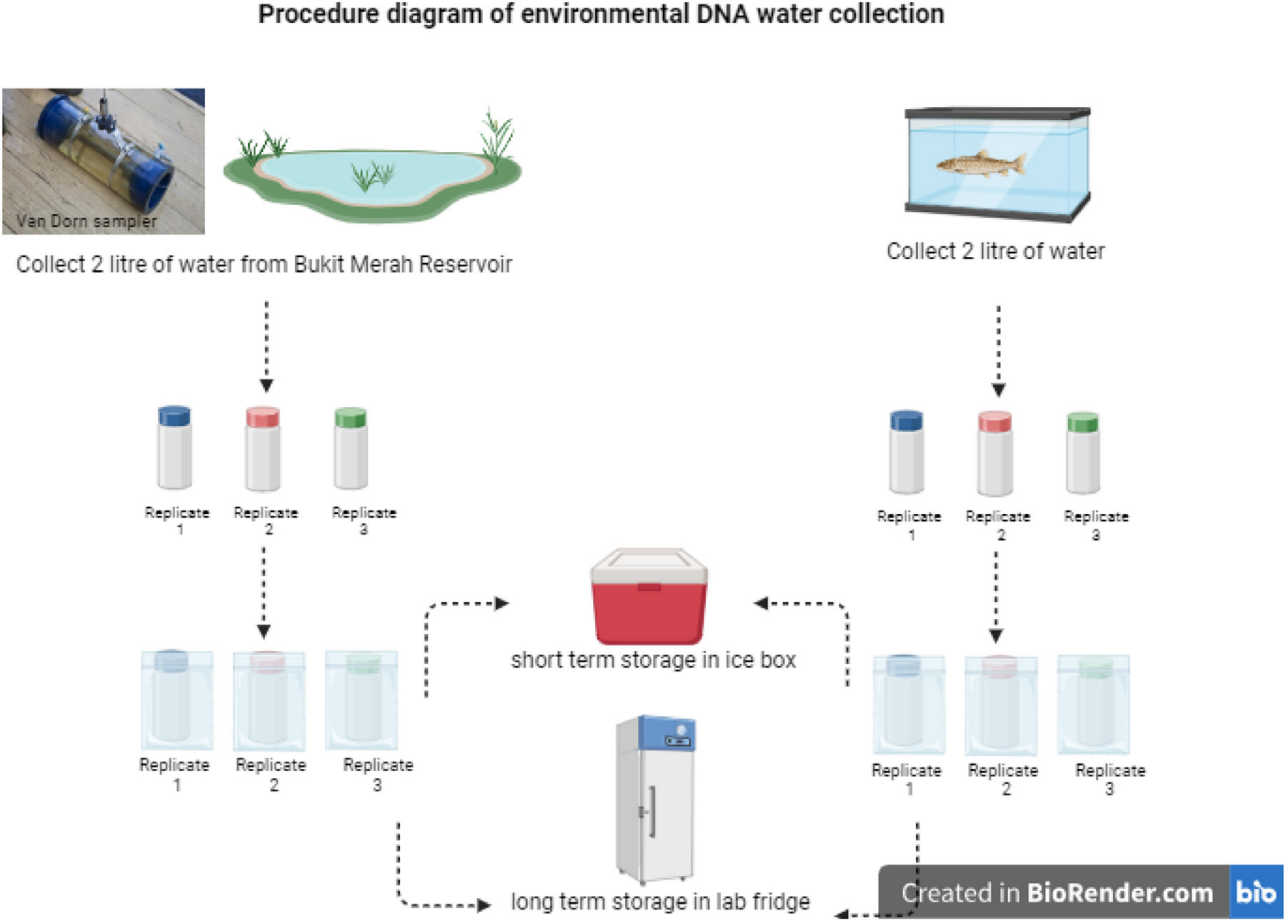
eDNA samples collection process.

Water samples for eDNA extraction were collected from the following sampling locations: (1) the location of the sighted and caught fish (B1 and B2), (2) the location of the sighted but uncaught fish (B3 and B4), and (3) the location of the fish that was neither sighted nor caught (B5 and B6), as shown in Table 2. For comparison and positive control, mesocosm study used aquarium water samples (with arowana inside) taken from a private arowana farm and designated as A1, A2, A3, A4, and A5 (Fig. 3).

**Table 2:** eDNA water collected (A1-A5 – aquarium tanks; B1-B6 – lake water) showing the occurence of Asian Arowana, *S. formosus.*

Location tagging ID	Water pH	Water temperature (°C)	Sighting/Presence of S. formosus
A1	6.90	25.2	Confirmed presence
A2	6.81	25.1	Confirmed presence
A3	6.83	25.3	Confirmed presence
A4	6.82	25.1	Confirmed presence
A5	6.88	25.0	Confirmed presence
B1	6.83	25.0	Sighted & caught
B2	6.96	24.9	Sighted & caught
B3	6.82	23.0	Sighted only
B4	6.87	23.6	Not sighted
B5	7.21	24.1	Sighted only
B6	7.22	25.0	Not sighted

Data on water temperature and pH were also collected to detect any components that could compromise the integrity of eDNA during sampling.

A consistent volume of 2 litres of water samples was filtered, to extract high-quality eDNA from water samples. Each of these 2 litres of water, which was then divided into three duplicates to minimise contamination. Samples from the lake water were collected using Van Dorn water sampler, securely linked to a rope marked with tape (to show the depth), and lowered 30 cm below the lake water level. The sampler was gradually removed from the water, and the water samples were transferred into labelled polypropylene bottles. Before opening, all bottles were cleaned with 10% bleach and wiped out with 70% ethanol. The water sampler was sanitised after each use with a towel soaked in 10% bleach and 70% alcohol. The bottles were then put in a marked zip lock bag and placed in an ice-filled cold box to retain the DNA integrity of the water samples and avoid deterioration. Water samples were kept at -20°C for later usage. Diagram of water collection procedures are as Fig. 3.

All of the objects and tools used in this sampling were pre-sanitised and labelled in the laboratory. To eliminate and limit the chance of contamination, all procedures were performed while wearing latex gloves and face masks.

### eDNA filtration

**Fig. 4:**
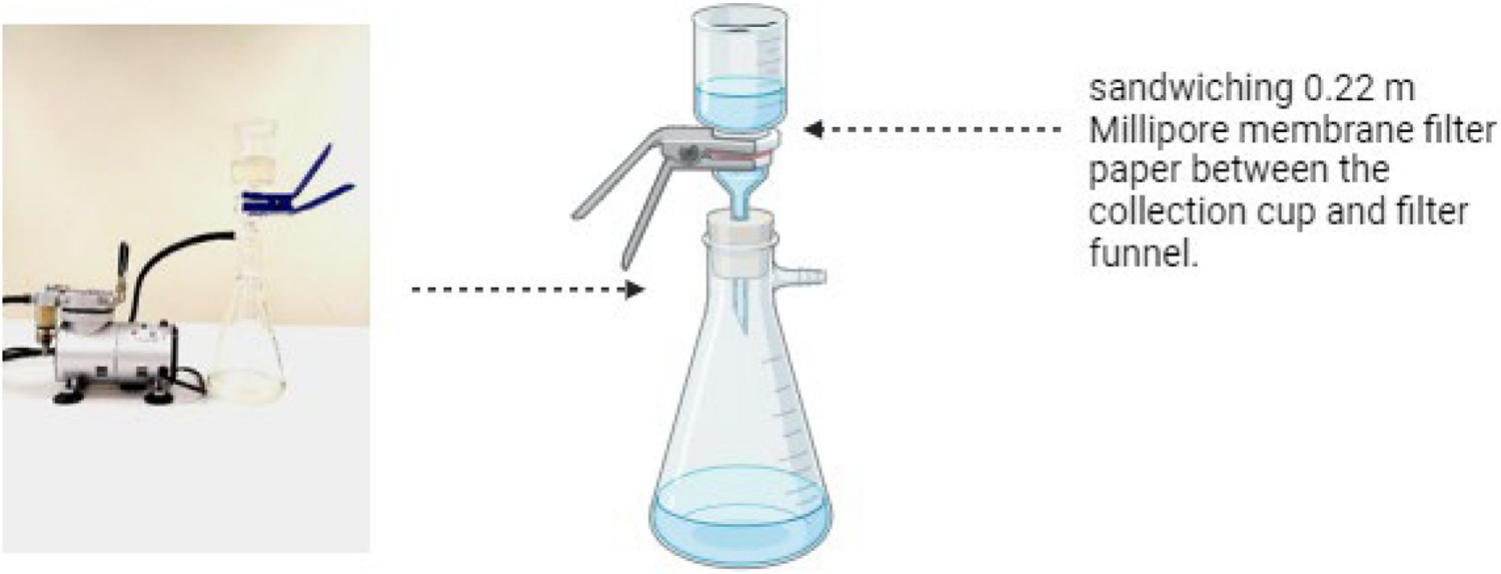
eDNA samples collection process.

Water samples were filtered as early as two days following collection. Water samples were evenly distributed among three distinct bottles, representing three replicates at each site. Before the filtration procedure, all equipment and the work environment were sterilized with a 10% bleach and 70% ethanol solution. The filtering equipment was set up as shown in Fig. 4, with the collecting cup and filter funnel linked to a vacuum source. Filtration was carried out by sandwiching 0.22 m Millipore membrane filter paper between the collection cup and filter funnel. The filtration procedure starts with filling the collecting cup with 250 mL of water samples and securing the equipment before initiating the vacuum water pump.

This technique was repeated for each sample, yielding a total of 2000 mL total water to be filtered. After filtering all of the water in the collection cup, the white filter membrane was grasped at opposing corners with two pairs of sterile forceps. The filter paper was rolled into a cylinder with the top pointing inward and inserted in a 5 mL Power Water DNA Bead Tube provided by the manufacturer of the Qiagen Power Water Kit (USA). The collection tube holding the filter paper was then labelled and kept at -20°C until DNA extraction was completed.

### *In vitro* specificity testing on environmental water samples

**Fig. 5:**
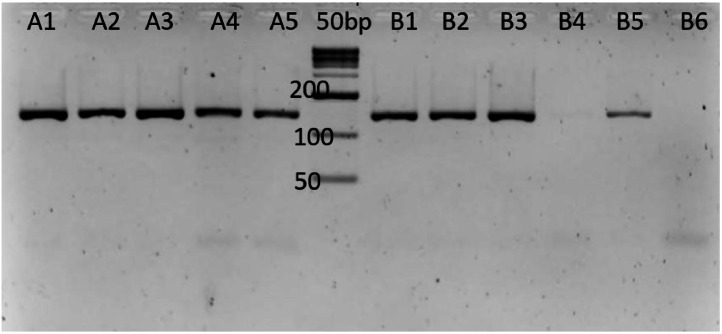
*In vitro* testing using S3 primers against extracted environmental DNA collected.

Amplification using conventional PCR was carried out on one replication of each extracted eDNA sample. The PCR reaction was performed in a 25L reaction mixture including 1X PCR Master Mix (Promega, USA) for S3 forward and reverse primers, as well as DNA template (120ng-500ng). A negative control reaction was also included, replacing ddH20 for the DNA template, whereas a positive control reaction used isolated *S. formosus* instead of eDNA. The PCR reactions were performed in a thermal cycler (T100 Bio-Rad, USA) with an initial denaturation step of 95°C for 2 minutes, followed by 35 cycles of 94°C for 30 seconds, an annealing step of 50°C for 45 seconds, an elongation step of 72°C for 90 seconds, and a final extension step of 72°C for 5 minutes. The PCR result (5 µL) was analysed using 2% agarose gel electrophoresis and 1 uL GelRed dye (Sigma-Aldrich, Germany). The gel image in Fig. 5 was obtained with the GeneSnap program (Syngene, UK).

Using S3 primer to test the positive controls from successfully amplified the control samples of eDNA from the *S. formosus* aquarium, whereas eDNA from the B1, B2, B3, and B5 locations at Bukit Merah Reservoir—where at least sightings of *S.formosus* were reported — also amplified. B4 and B6 sample findings were expected because there was no sighting or report of *S. formosus* during sample collection from that site. Arowana abundance and occurrence were reported to be distributed more likely in the Rasau (*Pandanus helicopus*) vegetation sites therefore it is highly possible to get eDNA traces of Arowana in areas nearby this kind of habitat.

## Conclusion

In this study, we designed a species-specific primer that can successfully detect the presence of *S. formosus* in environmental water samples from the Bukit Merah Lake. The detection of endangered *S. formosus* is possible through the application of eDNA tools, remarks the potential usage of eDNA in the conservation management of endangered and wildlife monitoring, in this case, the Asian golden arowana which is regulated through the CITES Act. In this case, the specific primer PCR techniques used to detect the presence of *S. formosus* which is a powerful tool for monitoring endangered or rare species in the wild, enabling researchers to track genetic diversity, population structure, and gene flow using non-invasive samples like mucus.

This technique provides critical insights into population health, habitat use, and migration, which are essential for developing effective conservation strategies such as protecting key habitats and managing breeding programs. The data generated can influence wildlife conservation policies by providing evidence for actions like creating protected with law enforcement or habitat restoration. Primer PCR also helps maintain genetic diversity in captive breeding programs, supports successful restocking efforts, and enables early detection of diseases, all of which are vital for the long-term survival of endangered species.

However, the eDNA primer for Asian Arowana has some limitations, including sensitivity to environmental factors, challenges in accurately reflecting population sizes due to DNA degradation, and difficulties in sampling from large or complex water bodies. Additionally, the relationship between eDNA concentration and population size is not fully understood, limiting its quantitative use. Future research should focus on conducting extensive field validation and developing better methods to quantify eDNA. Integrating eDNA with traditional monitoring techniques, expanding geographic studies, and encourage practical applications in conservation and law enforcement will enhance its effectiveness in protecting this endangered species.

## Ethics statements

All aquatic life examined in this study was already dead upon inspection, except for the scale sample taken from alive fish from the proposed Arowana sanctuary. Water samples were taken from aquarium water with living fish inside, carefully leaving the fish unharmed. We minimize the number of individuals used and adhere to all relevant legal and regulatory guidelines. Our research is transparent, ethically reviewed, and focused on long-term sustainability and positive conservation outcomes.All permission regarding the usage of endangered fish was granted by the Department of Fisheries, Malaysia.

## CRediT authorship contribution statement

**Nurul Affiqah Edora Mohd-Azhar:** Investigation, Writing – review & editing. **Haslawati Baharuddin:** Conceptualization, Supervision, Writing – review & editing, Validation. **Mohamad-Sufiyan Salmi:** Investigation, Writing – review & editing, Validation. **Noor Faizah Ismail:** Writing – review & editing, Validation. **Amatul-Samahah Md. Ali:** Writing – review & editing, Validation. **Syazwan Saidin:** Conceptualization, Supervision, Writing – review & editing, Validation. **Adibah Abu-Bakar:** Conceptualization, Supervision, Writing – review & editing, Validation.

## Declaration of competing interest

The authors declare that they have no known competing financial interests or personal relationships that could have appeared to influence the work reported in this paper.

## Data Availability

No data was used for the research described in the article.
